# Curcumin represses mouse 3T3-L1 cell adipogenic differentiation via inhibiting miR-17-5p and stimulating the Wnt signalling pathway effector Tcf7l2

**DOI:** 10.1038/cddis.2016.455

**Published:** 2017-01-19

**Authors:** Lili Tian, Zhuolun Song, Weijuan Shao, William W Du, Lisa R Zhao, Kejing Zeng, Burton B Yang, Tianru Jin

**Affiliations:** 1 Toronto General Research Institutes, University Health Network, Toronto, Canada; 2 Department of Physiology, University of Toronto, Toronto, Canada; 3 Banting and Best Diabetes Center, Faculty of Medicine, University of Toronto, Toronto, Canada; 4 Sunnybrook Research Institute, University of Toronto, Toronto, Canada

## Abstract

Understanding mechanisms underlying adipogenic differentiation may lead to the discovery of novel therapeutic targets for obesity. Wnt signalling pathway activation leads to repressed adipogenic differentiation while certain microRNAs may regulate pre-adipocyte proliferation and differentiation. We show here that in mouse white adipose tissue, miR-17-5p level is elevated after high fat diet consumption. miR-17-5p upregulates adipogenic differentiation, as its over-expression increased while its inhibition repressed 3T3-L1 differentiation. The *Tcf7l2* gene encodes a key Wnt signalling pathway effector, and its human homologue *TCF7L2* is a highly regarded diabetes risk gene. We found that *Tcf7l2* is an miR-17-5p target and confirmed the repressive effect of Tcf7l2 on 3T3-L1 adipogenic differentiation. The natural plant polyphenol compound curcumin possesses the body weight lowering effect. We observed that curcumin attenuated miR-17-5p expression and stimulated *Tcf7l2* expression in 3T3-L1 cells. These, along with the elevation of miR-17-5p expression in mouse epididymal fat tissue in response to high fat diet consumption, allowed us to suggest that miR-17-5p is among central switches of adipogenic differentiation. It activates adipogenesis via repressing the Wnt signalling pathway effector Tcf7l2, and its own expression is likely nutritionally regulated in health and disease.

Complicated cell signalling cascades are involved in cell fate determination in mammals, especially that of the mesenchymal origin.^1^ Mechanistic explorations of adipogenic differentiation, however, may lead to the recognition of novel therapeutic targets for obesity and its related metabolic diseases, including type 2 diabetes, fatty liver diseases, cardiovascular diseases and metabolic syndromes.^2, 3^ Following the discovery in the beginning of this century by Ross and colleagues that the canonical Wnt signalling cascade (defined as Wnt signalling hereafter) maintains pre-adipocytes in undifferentiated state via inhibiting the adipogenic transcription factors,^4^ numerous investigations have clarified that although Wnt signalling positively regulates osteogenesis, it inhibits adipogenesis.^5, 6^

TCF7L2 is an important component of the Wnt signalling cascade. It teams with free *β*-catenin (*β*-cat) molecule, forming the bipartite transcription factor *β*-cat/TCF, which serves as the key effector of the Wnt signalling. Since 2006, intensive genome-wide association studies (GWAS) have revealed that *TCF7L2* is among the type 2 diabetes risk genes,^7, 8^ while the expression of dominant negative TCF7L2 (TCF7L2DN) was shown to stimulate adipogenesis.^4^

During the last decade, numerous studies have revealed the regulatory effect of non-coding RNAs, including microRNAs on metabolic homeostasis.^9, 10, 11, 12^ As a recent study suggested that miR-17-5p represses another TCF member TCF7L1 in the human periodontal ligament stem cells^13^ and a previous study showed that over-expression of the whole miR-17/92 cluster stimulated 3T3-L1 adipogenic differentiation,^14^ we wonder whether miR-17-5p positively regulates adipogenesis via negatively regulating TCF7L2 and hence the Wnt signalling cascade.

Here we have tested this working hypothesis with the mouse pre-adipocyte cell line 3T3-L1 and revealed the role of miR-17-5p in stimulating adipogenesis and repressing Tcf7l2 expression with both gain-of-function and loss-of-function approaches. In addition, we have tested the effect of the plant polyphenol compound curcumin, which has been shown to repress adipogenesis,^15^ in this newly recognized signalling cascade. We observed that curcumin can repress miR-17-5p expression, resulted in increased Tcf7l2 expression and inhibited adipogenic differentiation.

## Results

### miR-17-5p positively regulates 3T3-L1 adipogenic differentiation

**Figure 1:**
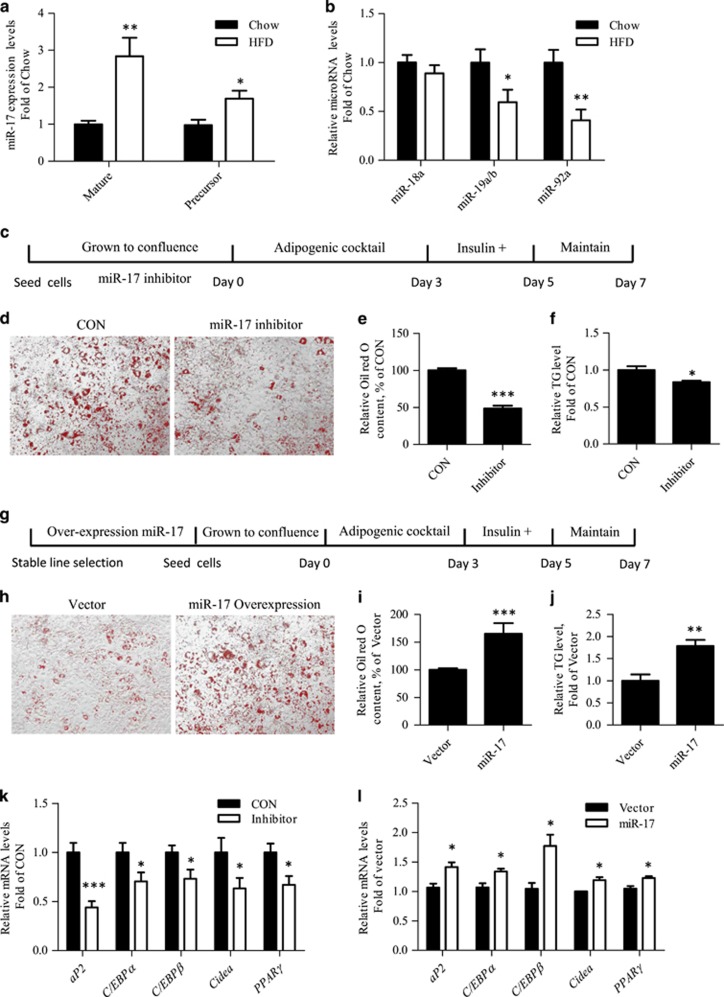
miR-17-5p activates 3T3-L1 adipogenic differentiation. (**a-b**) HFD feeding (12 weeks) increased the level of miR-17-5p but not levels of other miR-17-92 cluster members in mouse epididymal fat tissue. (**c**) Illustration of the differentiation procedure with miR-17-5p inhibition. (**d**) Oil red O staining. (**e**) Semi-quantitative assessment of Oil red O content. (**f**) Triglyceride levels in differentiated 3T3-L1 cells with and without miR-17-5p inhibition. (**g**) Illustration of the differentiation procedure with miR-17 overexpression. (**h**) Oil red O staining. (**i**) Semi-quantitative assessment of Oil red O content. (**j**) Triglyceride levels in differentiated 3T3-L1 cells with and without miR-17 over-expression. (**k-l**) Detection of expression of genes that encode five adipogenic differentiation markers with and without miR-17-5p inhibition (**k**), as well as with and without miR-17 over-expression (**l**). *N*> or=3 for panels A, B, D–F and H–L. *P<0.05; **P<0.01; ***P<0.001. CON, control; miR-17, miR-17-5p

A previous study demonstrated that the transfection of the whole miR-17/92 cluster accelerated 3T3-L1 adipogenic differentiation.^14^ Here we have first of all tested whether high fat diet (HFD) feeding affects the expression of members of this cluster in the mouse epididymal fat tissue. As shown in Figures 1a and b, 12-week HFD feeding significantly increased the expression level of miR-17-5p, but not the other cluster members. In addition, the stimulatory effect of HFD on miR-17-5p expression was not observed in mouse liver (Supplementary Figures 1A–B). HFD feeding also did not affect expression of other members of the miR-17/92 cluster in the mouse liver (Supplementary Figure 1C).

We then tested the effect of miR-17-5p inhibition on 3T3-L1 adipogenic differentiation (Figure 1c). This inhibition protocol significantly reduced the miR-17-5p expression level (Supplementary Figure 2A). Oil Red O (ORO) staining was performed at day 7 (Figure 1d). Figure 1e shows the result of our semi-quantitative analyses of ORO staining, and Figure 1f shows that miR-17 inhibition reduced the triglyceride level in differentiated 3T3-L1 cells. These observations collectively indicate that miR-17-5p inhibition repressed 3T3-L1 adipogenic differentiation. To test the effect of miR-17-5p over-expression, a stable cell selection procedure with puromycin was utilized for either control vector or the miR-17 precursor expressing vehicle was included; as the DNA transfection efficiency in this cell line was relatively low (Figure 1g). Supplementary Figure 2B shows the elevation of miR-17 level in 3T3-L1 cells after the miR-17 vehicle transfection. Figures 1h and i show that differentiated 3T3-L1 cells with miR-17 over-expression exhibited elevated ORO staining, comparing with cells transfected with the same amount of the control vector and underwent the same stable-cell selection procedure. Figure 1j shows that miR-17 over-expression increased the cellular triglyceride level in differentiated 3T3-L1 cells. Figures 1k and l show that miR-17 inhibition (K) and over-expression (L) generated opposite effects on mRNA levels of five genes that encode adipogenic differentiation markers, including aP2 (adipocyte fatty acid-binding protein), C/EBP*α* (CCAAT/enhancer-binding protein *α*), C/EBP*β* (CCAAT/enhancer-binding protein *β*), Cidea (cell death-inducing DFFA-like effector a) and PPAR*γ* (peroxisome proliferator-activated receptor *γ*). We hence suggest that miR-17-5p positively regulates 3T3-L1 differentiation.

### Tcf7l2 is likely a direct downstream target of miR-17-5p

**Figure 2:**
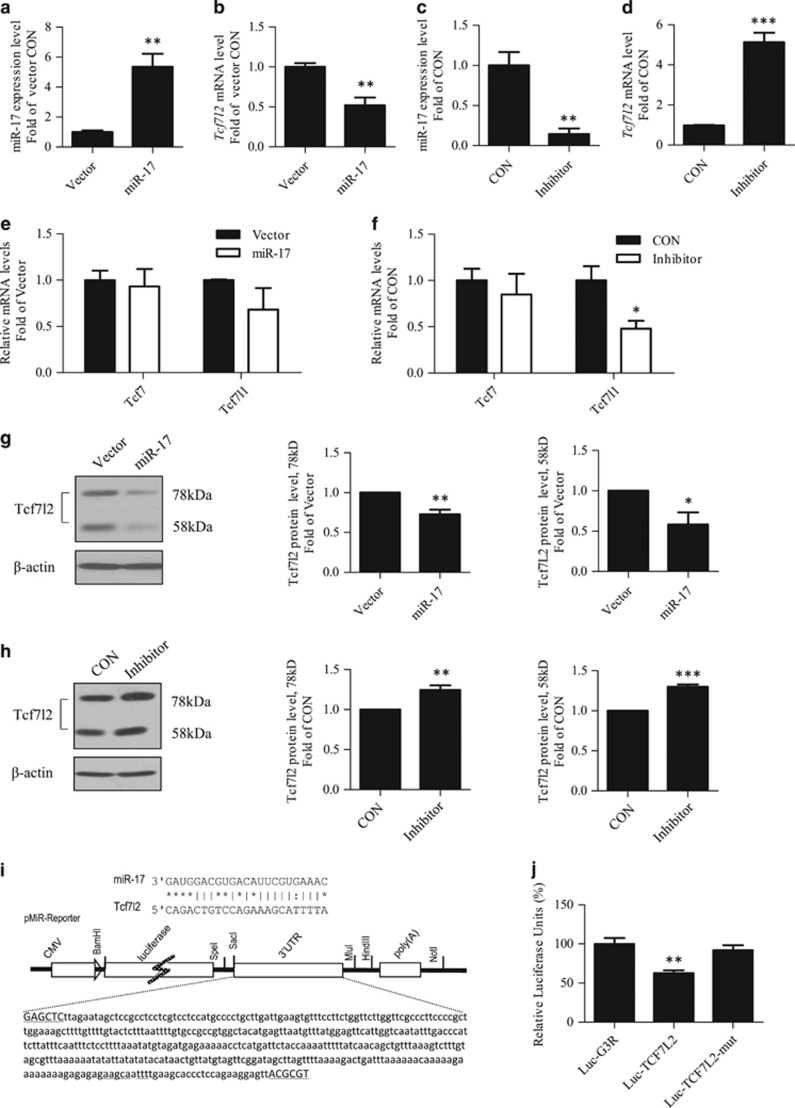
Tcf7l2 level is negatively regulated by miR-17-5p in 3T3-L1 cells. (**a-d**) miR-17 over-expression repressed, while miR-17-5p inhibition increased *Tcf7l2* levels. (**e-f**) miR-17 over-expression generated no effect on Tcf7 or Tcf7l1 expression (**e**), while its inhibition reduced Tcf7l1 expression (**f**). (**g**) miR-17 over-expression repressed Tcf7l2 protein levels. The middle and right panels show the results of densitometric analyses. (**h**) miR-17-5p inhibition increased Tcf7l2 protein levels. For (**g**) and (**h**), middle and right panels show the results of densitometric analyses. (**i**) Illustration of the potential miR-17-5p binding site on *Tcf7l2* 3′UTR. (**j**) Decreased LUC activities were observed in cells co-transfected with miR-17 and Luc-T*cf7l2*, which was reversed when the miR-17-5p binding site was mutated. *N*> or =3 for panels (**a–h**) and (**j**). *P<0.05; **P<0.01; ***P<0.001. CON, control; miR-17, miR-17-5p

We then directly tested the effect of miR-17-5p on Tcf7l2 expression in undifferentiated 3T3-L1 cells. The miR-17 vehicle transfection increased miR-17-5p levels (Figure 2a), associated with reduced *Tcf7l2* mRNA levels (Figure 2b). The inhibition of miR-17-5p, however, resulted in a significant increase on *Tcf7l2* mRNA level (Figures 2c and d). Such regulations were not observed for Tcf7 or Tcf7l1 (Figure 2e and f). In mouse primary hepatocytes, miR-17 over-expression and miR-17-5p inhibition also generated repressive and stimulatory effect on *Tcf7l2* mRNA expression, respectively (Supplementary Figure 3).

Western blotting was then performed in undifferentiated 3T3-L1 cells with miR-17 over-expression or miR-17-5p repression. miR-17 over-expression reduced the levels of the two major Tcf7l2 isoforms (78 and 58 kDa) (Figure 2g). miR-17-5p inhibition, however, generated the opposite effect (Figure 2h).

A potential miR-17-5p binding motif was located within the mouse *Tcf7l2* 3'UTR (Figure 2i). Luciferase-reporter constructs were generated for testing whether this motif potentially affect miR-17-5p-mediated gene expression. As shown in Figure 2j, in response to miR-17 co-transfection, the insertion of the mouse *Tcf7l2* 3′UTR reduced the luciferase activity when compared with the parental Luc-G3R construct; while such repression was abolished when the located miR-17-5p binding site was mutated. Thus, Tcf7l2 is likely a direct downstream target of miR-17-5p.

### Tcf7L2 negatively regulates 3T3-L1 adipogenic differentiation

**Figure 3:**
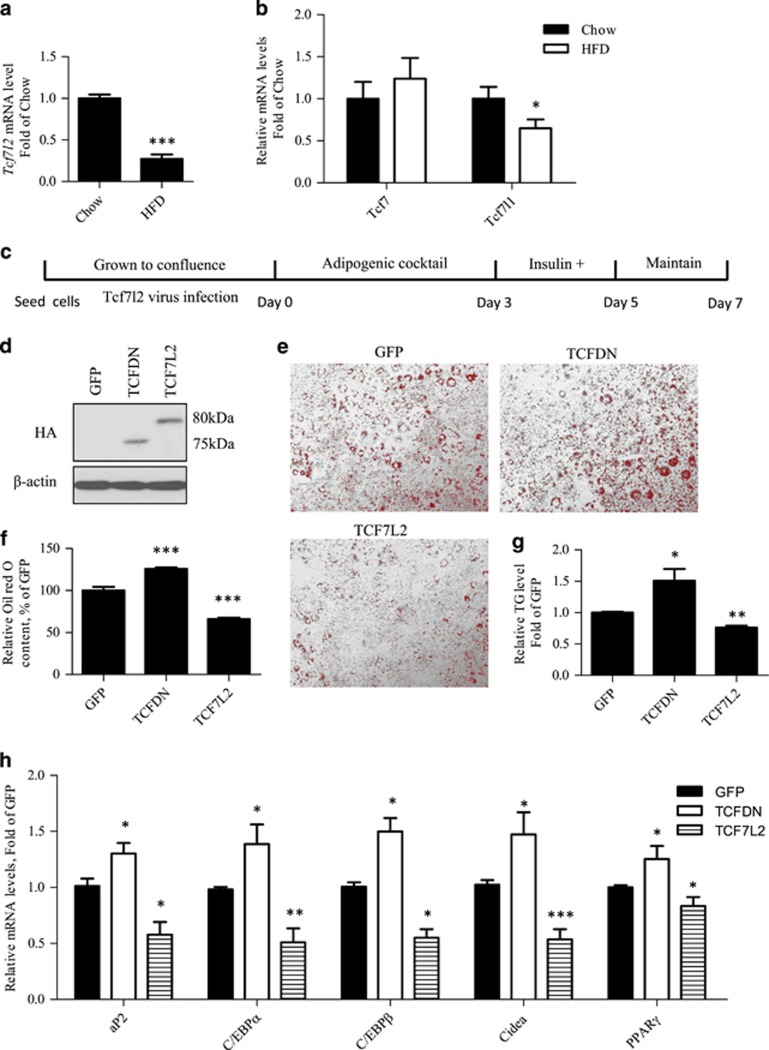
Tcf7l2 negatively regulates 3T3-L1 differentiation. (**a-b**) HFD feeding decreased *Tcf7l2* and *Tcf7l1* but not *Tcf7* mRNA levels. (**c**) Illustration of the differentiation procedure with virus infection. (**d**) Detection of exogenous expression of TCF7L2 and TCF7L2DN in 3T3-L1 cells after virus infections for 48 h by western blotting with the HA-tag antibody. (**e-f**) Oil red O staining and semi-quantitative assessment of Oil red O content. (**g**) Triglyceride levels in differentiated 3T3-L1 cells with indicated virus infection. (**h**) Detection of genes that encode five adipogenic differentiation markers in differentiated 3T3-L1 cells infected with the correspondent adenovirus. *N*> or =3 for panels (**a**), (**b**) and (**e–h**). *P<0.05; **P<0.01; ***P<0.001. TCFDN, Ad-TCF7L2DN; miR-17, miR-17-5p

A previous study showed that expression of TCF7L2DN stimulates 3T3-L1 differentiation.^4^ To further determine the patho-physiological relevance of Tcf7l2, we assessed the effect of HFD on expression of Tcf members in the visceral fat tissue. As shown in Figures 3a and b, HFD feeding reduced both *Tcf7l2* and *Tcf7l1*, but not *Tcf7* expression in the mouse epididymal fat tissue.

The possession of HA-tagged adenovirus vehicles for wild type TCF7L2 and TCF7L2DN^16^ allowed us to assess the effect of their expression on 3T3-L1 differentiation (Figure 3c). Figure 3d shows that 48 h after the virus infection in 3T3-L1 cells, we were able to detect the expression of HA-tagged exogenous TCF7L2 or TCF7L2DN. Figures 3e and g confirm that TCF7L2DN expression increased 3T3-L1 differentiation while wild type TCF7L2 expression reduced 3T3-L1 differentiation. The opposite effects of Ad-TCF7L2 and Ad-TCF7L2DN infection on mRNA levels of a battery of adipogenic genes in differentiated 3T3-L1 cells are shown in Figure 3h.

### The repressive effect of Ad-TCF7L2 infection can be attenuated by miR-17 over-expression

**Figure 4:**
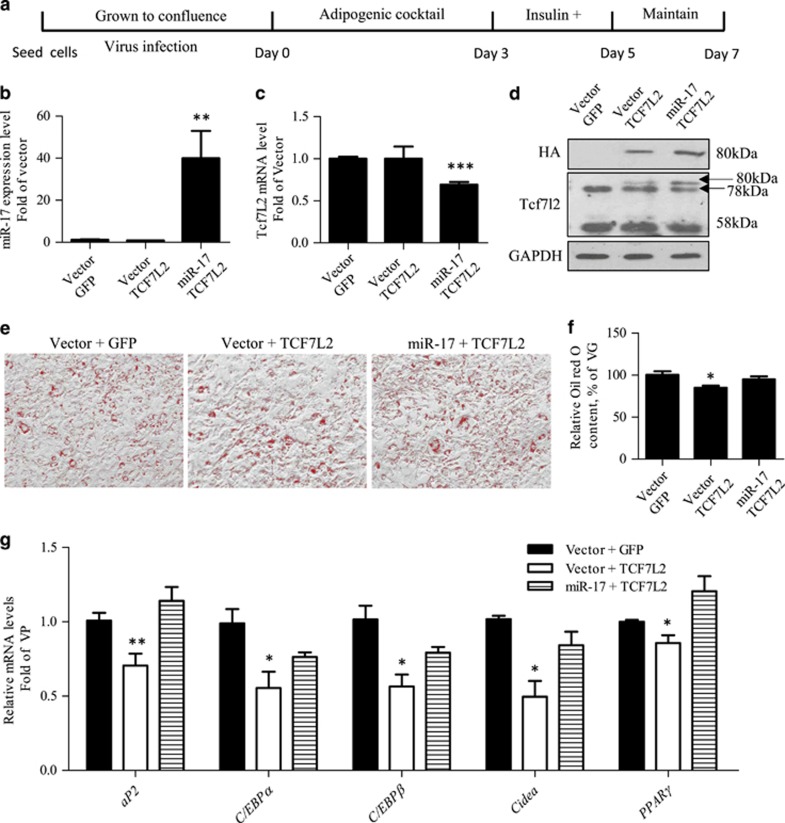
The repressive effect of TCF7L2 over-expression on adipogenic differentiation can be blocked by miR-17 over-expression. (**a**) Illustration of the differentiation procedure with virus infection. (**b–d**) Detection of miR-17-5p (**b**), endogenous *Tcf7l2* mRNA (**c**), as well as exogenous and endogenous TCF7L2/Tcf7l2 (**d**) in indicated virus infected 3T3-L1 cells 24 h after virus infection. (**e**–**f**) Oil red O staining and semi-quantitative assessment of Oil red O content. (**g**) Detection of five genes that encode adipogenic differentiation markers in differentiated 3T3-L1 cells infected with the correspondent virus. *N*> or =3 for panels (**b**–**c**) and (**e**–**g**). *P<0.05; **P<0.01; ***P<0.001; miR-17, miR-17-5p

We then assessed whether the repressive effect of TCF7L2 over-expression on 3T3-L1 adipogenic differentiation can be reversed by miR-17 over-expression. For this purpose, 3T3-L1 cells were infected by either the control virus, or Ad-TCF7L2, or Ad-TCF7L2 and the miR-17 lentivirus (Figure 4a). The stimulatory effect of miR-17 over-expression on miR-17-5p and its repressive effect on endogenous *Tcf7l2* mRNA level were shown in Figures 4b and c, while Figure 4d shows the detection of exogenous TCF7L2 (80 kDa) and endogenous Tcf7L2 (78 and 58 kDa) in cells infected with the control virus, or Ad-TCF7L2, or Ad-TCF7L2 and miR-17 lentivirus. As shown in Figures 4e and f, the repressive effect of TCF7L2 on adipogenic differentiation was blocked with miR-17 over-expression. Figure 4g shows the correspondent alterations on adipogenic gene expression in 3T3-L1 cells infected with the control virus, or Ad-TCF7L2, or Ad-TCF7L2 and miR-17.

### Curcumin represses 3T3-L1 adipogenic differentiation, associated with miR-17-5p repression and Tcf7l2 stimulation

**Figure 5:**
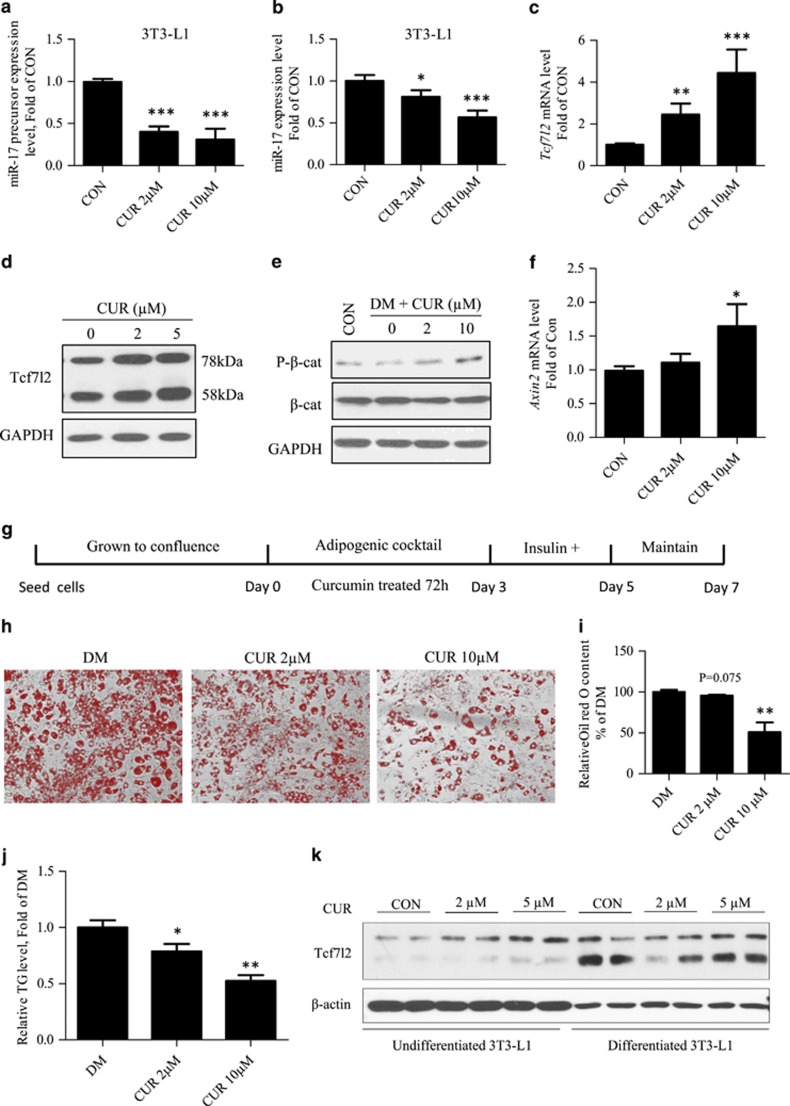
Curcumin represses 3T3-L1 cell differentiation, associated with the repression of miR-17-5p and the stimulation of Tcf7l2. (**a–b**) Curcumin treatment (with indicated dosage for 6 h) repressed miR-17 precursor and mature miR-17-5p. (**c**–**d**) Tcf7l2 mRNA and protein levels were elevated in undifferentiated 3T3-L1 cells after 6-h curcumin treatment. (**e**) Curcumin treatment stimulated *β*-cat Ser675 phosphorylation in undifferentiated 3T3-L1 cells. (**f**) Curcumin treatment increased *Axin2* expression in 3T3-L1 cells. (**g**) Illustration of the differentiation procedure with curcumin treatment. (**h**–**i**) Oil red O staining and semi-quantitative assessment of Oil red O content. (**j**) Triglyceride levels in differentiated 3T3-L1 cells with and without curcumin treatment. (**k**) Tcf7l2 protein levels in un-differentiated or differentiated 3T3-L1 cells after the treatment with indicated dosages of curcumin for 24 h. *N*> or =3 for panels (**a**–**c**), (**f**), (**h**–**j**). *P<0.05 **P<0.01; ***P<0.001. CON, control; CUR, curcumin; DM, differentiation medium only; miR-17, miR-17-5p

The *in vivo* body weight lowering effect of curcumin and its repressive effect on 3T3-L1 differentiation have been previously demonstrated by our team and by others.^15, 17, 18^ However, no investigations have addressed the involvement of Tcf7l2 or miR-17-5p in response to curcumin treatment. We hence conducted 3T3-L1 differentiation experiment in the presence and absence of curcumin. Before doing so, we tested the effect of curcumin on miR-17-5p and Tcf7l2 expression in undifferentiated 3T3-L1 cells. As shown in Figures 5a and b, curcumin treatment (2 μM or 10 μM for 6 h) repressed miR-17-5p precursor or mature miR-17-5p levels. In addition, when rat mature adipocytes were treated with curcumin for 6 h, miR-17-5p level was also significantly repressed, associated with increased Tcf7l2 protein levels (Supplementary Figure 4). Repressed miR-17-5p expression in response to curcumin treatment was associated with increased Tcf7l2 expression at mRNA and protein levels in 3T3-L1 cells (Figures 5c and d). Figure 5e shows that treating 3T3-L1 cells with curcumin-containing differentiation medium increased *β*-cat S675 phosphorylation, an event that is positively associated with Wnt signalling cascade activation.^19^ Furthermore, curcumin treatment increased the expression of the Wnt pathway downstream target Axin2 (Figure 4f). Figure 5g shows our 3T3-L1 differentiation procedure with curcumin treatment. Evidently, treating 3T3-L1 cells with 10 μM curcumin during the first 72 h of the differentiation procedure significantly repressed the differentiation (Figures 5h–j). Figure 5k shows that 24-h curcumin treatment increased Tcf7l2 protein expression, especially the 78-kDa isoform in samples collected at undifferentiated condition. In differentiated 3T3-L1 cells, 24-h curcumin treatment generated no appreciable stimulatory effect. Furthermore, differentiated 3T3-L1 cells show elevated TCF7L2 expression, especially the 58 kDa isoform, regardless of curcumin treatment.

It is worth to mention that Rb2 is a previously identified target of the miR-17/92 cluster.^14^ We show here that Rb2 expression was also stimulated by miR-17-5p inhibition (Supplementary Figure 5A) and inhibited by miR-17 overexpression (Supplementary Figure 5B). The expression of Rb2, however, was not upregulated by curcumin in undifferentiated 3T3-L1 cell line (Supplementary Figure 5C). Furthermore, it appears that Rb2 mRNA expression cannot be stimulated by HFD feeding (Supplementary Figure 5D).

**Figure 6:**
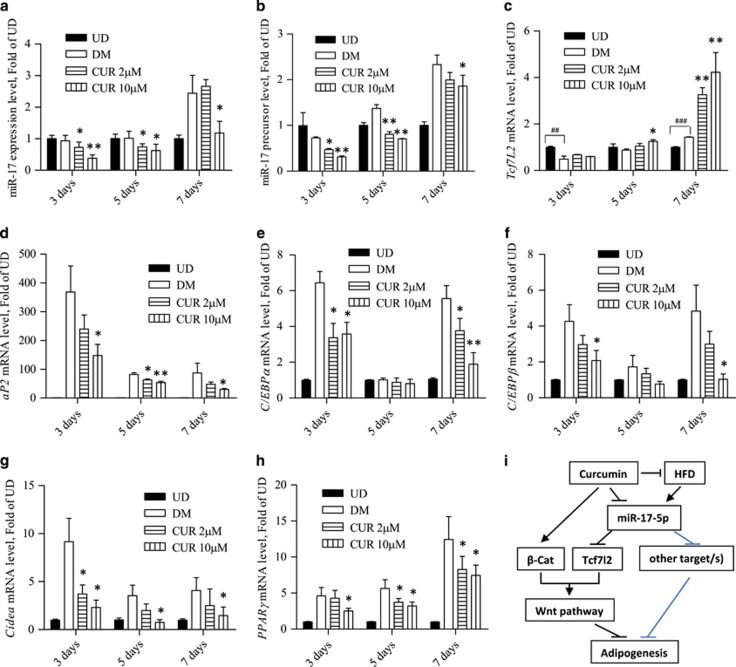
Repressed 3T3-L1 differentiation with curcumin treatment is associated with reduced miR-17-5p and increased Tcf7l2. (**a-h**) qRT-PCR showed the results of indicated target gene in 3T3-L1 cells collected at indicated time intervals during the differentiation process. (**i**) Illustration of the regulatory network on curcumin-mediated repression of adipogenesis, involving miR-17-5p inhibition and Wnt signalling cascade activation. *N*> or =3 for panels (**a**–**i**); *P<0.05; **or ^##^*P*<0.01; ***P<0.001. * or **, when curcumin treated group was compared with the DM group. ##, when the DM group was compared with the UD group. UD, undifferentiated; CUR, curcumin; DM, differentiation medium only; miR-17, miR-17-5p

Cell samples from the above differentiation experiment (Figure 5g) were collected at days 3, 5 and 7 for RNA extract and qRT-PCR (Figure 6). Repressed miR-17-5p (Figure 6a) or its precursor (Figure 6b) expression in response to curcumin treatment was appreciable at days 3 and 5 at both dosages. At day 7, the repression was appreciable only when the dosage of curcumin reached 10 *μ*M. Significant stimulation on *Tcf7l2* expression by curcumin treatment, however, was observed at day 5 with 10 μM curcumin and at day 7 with both dosages (Figure 6c). Figures 6d–h shows the detection of a battery of genes that encode adipogenic differentiation markers. Briefly, elevated expression of aP2, C/EBP*α*, C/EBP*β*, Cidea and PPAR*γ* in response to the differentiation was blocked by curcumin treatment.

## Discussion

We have learned for more than a decade that Wnt pathway activation leads to repressed adipogenic differentiation,^4^ while dietary curcumin consumption can lower body weight gain in HFD fed mice^17, 18^ and prevent type 2 diabetes development in the pre-diabetic population.^20^ The mechanistic linkage between Wnt activation and curcumin treatment has not been investigated until recently. Ahn *et al* reported that curcumin repressed 3T3-L1 differentiation, associated with increased expression of Wnt10b, Wnt receptor Ftz2 and the co-receptor LRP5.^15^ Ahn and colleagues,^15^ however, did not examine the Wnt pathway effector Tcf7l2, of which the human homologue is an important diabetes risk gene.^7, 8^ Based on a recent study that miR-17-5p repressed another TCF member TCF7L1 in another cell lineage^13^ and a previous investigation that miR-17/92 cluster over-expression stimulated 3T3-L1 adipogenic differentiation,^14^ we tested our hypotheses that miR-17-5p positively regulates adipogenesis via negatively regulating TCF7L2, and that curcumin can restore the Wnt activity and hence represses adipogenesis.

Our pre-test showed that the dosage of curcumin utilized by Ahn *et al*^15^ (25 μM) resulted in intensive cell death and hence reduced the dosages to 2–10 *μ*M. Such dosages, in our hand, generated no substantial morphological changes of the 3T3-L1 cells (Supplementary Figure 6). With our experimental settings, we demonstrated the stimulatory effect of curcumin on Tcf7l2 mRNA and protein expression and *β*-cat S675 phosphorylation, associated with reduced miR-17-5p level and increased expression of the Wnt target Axin2. Thus, our current investigation revealed the existence of a regulatory network that controls adipogenic differentiation, involving dietary polyphenol consumption, Wnt signalling pathway activation and microRNA regulation.

Figure 6i summarized our current understanding. Briefly, HFD consumption may upregulate adipogenic differentiation via increasing the expression of miR-17-5p, which downregulates Tcf7l2 expression and the Wnt signalling cascade. Curcumin, however, can reduce the miR-17-5p level, releasing its repression on Tcf7l2. Furthermore, curcumin treatment activates the Tcf co-factor *β*-cat via facilitating its S675 phosphorylation. Although the mechanism underlying the stimulation of *β*-cat S675 phosphorylation in adipogenic cells by curcumin is yet to be further explored, this could be attributed to its insulin signalling sensitization effect, demonstrated by our team in hepatocytes.^21^ Evidently, insulin can stimulate *β*-cat S675 or S552 phosphorylation in a battery of cell lineages.^8, 22, 23, 24, 25^ It should be emphasized here that curcumin can regulate many cellular events while miR-17-5p may have numerous downstream targets. Detailed *in vivo* contributions of the signalling events listed in this figure needs further investigations.

Non-coding RNAs including microRNAs play important roles in various biological activities including metabolic homeostasis.^10, 11^ Previous investigations have implicated a number of microRNAs in regulating adipogenic differentiation, including the miR-17/92 cluster.^14, 26, 27, 28^ The organization of members of this cluster is highly conserved among humans and rodents.^29^ Members of this cluster are involved in turmorigenesis, embryonic development, immune diseases, cardiovascular diseases, neurodegenerative diseases and ageing. Targeted deletion of this cluster in mice resulted in perinatal lethal,^30^ while its overexpression in intestinal epithelial cells decreased the tumour size in a chemical-induced colorectal cancer mouse model.^31^

Wang *et al.* found that over-expression of the whole miR-17/92 cluster in 3T3-L1 cells accelerated their adipogenic differentiation, and their mechanistic exploration implicated Rb2 repression.^14^ Here we located the stimulatory effect of this cluster to its first member miR-17, although we cannot eliminate the involvement of other members. Nevertheless, mouse visceral adipose tissue expression of miR-17-5p, but not the other cluster members, was shown to be stimulated by HFD feeding. It is worth to mention here that recently, Li *et al.* reported that in human adipocyte-derived mesenchymal stem cells, BMP2 is a direct target of miR-17-5p and miR-106a, while BMP2 knockdown repressed osteogenesis but increased adipogenesis.^32^ A recent study demonstrated the effect of curcumin on attenuating BMP2 expression in vascular smooth muscle cells.^33^ It remains to be determined whether BMP2 can be repressed by curcumin in the 3T3-L1 cell differentiation model. Nevertheless, the above observations are consistent with our finding that miR-17-5p stimulates adipogenic differentiation.

In agreement with Wang *et al.*^14^ we found that Rb2 is likely a target of miR-17-5p, as its expression can be stimulated by miR-17-5p inhibition but inhibited by miR-17 overexpression. Its expression in undifferentiated 3T3-L1 cells, however, cannot be stimulated by curcumin (Supplementary Figure 5). As the repressive effect of exogenous TCF7L2 over-expression on 3T3-L1 adipogenic differentiation can be reversed by miR-17 over-expression, it is likely that *β*-cat/Tcf7l2 is not the solo target of miR-17-5p in facilitating adipogenic differentiation. Further investigations are needed to determine how the repression of Tcf7l2, Rb2 and other miR-17-5p targets collectively contribute to the facilitation of adipogenesis in response to miR-17-5p elevation. It is also necessary to point out that in response to HFD feeding, increased miR-17-5p level was observed in the white adipose tissue but not in the liver, although pathological effects of miR-17-5p in the liver have been documented.^34, 35^ Thus, if miR-17-5p is a central switch in response to HFD consumption, adipose tissue could be a more sensitive organ.

How curcumin represses miR-17-5p expression remains to be explored. A PPAR response element was located within the promoter region of the miR-17/92 cluster and we found previously that in the liver, PPAR*α* is a miR-17-5p target.^34^ PPRA*α* over-expression, however, stimulated miR-17-5p expression, indicating the existence of complicated reciprocal regulation.^34^

miR-17 precursor can produce two functional regulatory microRNAs, miR-17-5p and miR-17-3p. Here we focussed on miR-17-5p as its metabolic role has been demonstrated in several previous studies. We found that miR-17-5p promotes chemotherapeutic drug resistance and colon cancer metastasis, and that fatty liver development induced by dexamethasone injection in mice can be attenuated by reducing miR-17-5p levels.^34, 35^ Recently, Jacovetti *et al* demonstrated that microRNAs including miR-17-5p and miR-181b play a central role in postnatal *β*-cell maturation.^10^ Due to a technical issue we cannot eliminate the involvement of miR-17-3p in our over-expression experiment; the inhibitor utilized in this study, however, was specific for miR-17-5p. Furthermore, for each of the experiments with miR-17 over-expression, the elevation of miR-17-5p was confirmed by qRT-PCR. Another technical drawback in our study is the use of the 3T3-L1 cell line. The degree of 3T3-L1 adipogenic differentiation can be influenced by both DNA transfection and virus infection. Nevertheless, proper controls have been included in each set of the cell differentiation experiments.

Following the discovery by GWAS that TCF7L2 is a type 2 diabetes risk gene,^7^ its function in various metabolic organs has been intensively investigated.^8^ In human adipose tissue, TCF7L2 expression and alternative splicing were shown to be regulated by health and disease, as well as by weight loss and exercise.^36, 37, 38, 39, 40^ Adipocyte-derived Wnt signalling molecules were also suggested to function as endocrine-like factors in stimulating *β*-cell insulin secretion.^41^ It remains to be determined whether miR-17-5p plays a role in human adipogenesis, involving TCF7L2 expression and alternative splicing. The exon-intron organizations of human TCF7L2 and mouse Tcf7l2 are highly conserved. For each of the two species, all 13 alternatively spliced isoforms contain the same exon 17 sequence, which covers the 3′UTR region for each of the isoforms.^42, 43^ A proper human cell model is desired for conducting further examinations.

A paradoxical observation in this study is that although the repression of curcumin on 3T3-L1 differentiation was associated with repressed miR-17-5p expression and increased Tcf7l2 expression, basal Tcf7l2 level in the absence of curcumin treatment was not reduced after the differentiation. Although this appears to be contradictory with the theory that Wnt signalling negatively regulates adipogenesis,^4^ we present our interpretations on this matter as follows. First, the function of Tcf7l2, or other Tcf members, is bi-directional.^8^ How does it regulate Wnt target gene expression is dependent on the availability and phosphorylation status of *β*-cat. The expression of Tcf7l2 itself during 3T3-L1 differentiation cannot be simply considered as a positive entity of Wnt activation, unless its co-factor, *β*-cat, is also activated. Second, it was the 58-kDa isoform of Tcf7l2 but not the 78 kDa one that was substantially increased after 3T3-L1 differentiation (Figure 5k). Whether different isoforms of Tcf7l2 exert different or even opposite regulatory functions on adipogenesis or other cellular events remain to be determined.

The metabolic beneficial effect of brown adipocytes and white adipocyte browning have received broad attention recently.^2^ An early investigation showed that Wnt signalling also represses the genesis of brown adipocytes while Wnt activation in mature brown adipocytes stimulated their conversion into white adipocytes.^44^ Whether the regulatory network revealed in this study functions the same or different in brown adipocytes is worth to be investigated.

In summary, our current study revealed the positive effect of miR-17-5p on white adipocyte differentiation. This is at least partially achieved via targeting the Wnt signalling cascade effector Tcf7l2. Our investigation has also expanded the mechanistic understanding on the metabolic beneficial effects of the curry compound curcumin into the control of miRNA levels and the stimulation of the Wnt signalling cascade.

## Materials and methods

### Animals

Male C57BL/6 mice were utilized for HFD feeding and epididymal fat as well as liver tissue collection as we have previously described.^17, 45^ Briefly, 5-week-old male C57BL/6 mice were purchased from the Charles River Laboratories (Montreal, Canada). After a 1-week acclimatization period, the mice were randomly divided to two groups with six mice per group: the chow diet group and the HFD group. At the end of the 12 week with the correspondent diet feeding, increased body weight gain and impaired glucose tolerance in response to HFD consumption were verified with routine methods.^17^ Mice were then scarified for epididymal adipose tissue and liver tissue collections.

Six-week-old male Wistar rats, purchased from the Charles River Laboratories, were utilized for mature white adipocyte isolation.^46^ Briefly, epididymal fat pads from the Wistar rats were removed and rinsed with PBS immediately after the animals were killed. The fat tissues were then minced, followed by collagenase digestion (37 °C for 1 h). Undigested tissue fragments were removed via a filter procedure, while mature adipocytes were obtained with a centrifugation procedure for collecting the supernatant layer.

All animals were maintained at relative humidity of 50% and room temperature, under a 12-h light/dark cycle with free access to both food and water. The protocols for animal use and euthanasia were approved by the University Health Network Animal Care Committee and were conducted in accordance with the guidelines of the Canadian Council of Animal Care.

### Chemicals

3-isobutyl-1-methylxanthine (IBMX) and curcumin were purchased from Sigma Aldrich (Oakville, Ontario, Canada). Sodium dexamethasone was the product of Omega Laboratories Ltd (Montreal, Canada). Insulin was a gift from Novo Nordisk (Mississauga, Canada). miR-17 inhibitor was the product of Shanghai GenePharma Co, Ltd (Shanghai, China).^47^

### 3T3-L1 adipogenic differentiation

The 3T3-L1 cell line was purchased from American Type Culture Collection. Cells were cultured in DMEM containing 10% fetal bovine serum (FBS, Sigma Aldrich). The differentiation procedure was initiated 2 days after cells reached 100% confluence by adding the differentiation cocktail (0.5 mM IBMX, 4 *μ*g/ml dexamethasone and 10 *μ*g/ml insulin in DMEM containing10% FBS). IBMX and dexamethasone were then removed at the end of day 3, while insulin was maintained for additional two days. Thereafter, cells were grown in the maintaining medium (DMEM with 10% FBS).

ORO staining has been reported previously.^46^ Briefly, the differentiated 3T3-L1 cells were fixed with 10% formalin for 1 h and washed four times with water. The cells were then rinsed with 60% isopropanol for 5 min followed by the incubation with freshly prepared ORO working solution for 15 min. After four times washing with water, images were acquired under the light microscope. ORO was then extracted from fixed cells with 100% isopropanol, followed by OD values determination at wavelength of 500 nm. Relative ORO content was presented as percentage with the correspondent control as 100%, normalized against the protein content. Triglyceride measurement was performed by extracting cellular triglyceride with 5% NP-40. The triglyceride content was measured with the Triglyceride Determination Kit (Sigma TR0100). Relative triglyceride levels were presented as fold changes to the correspondent control, normalized against the protein content.

### Gene transfection, virus infection, real-time PCR and western blotting

miR-17 or its inhibitor transfection were performed with Lipofectamine. The generation of Ad-TCF7L2, Ad-TCF7L2DN and miR-17 over-expression plasmid has been previously presented.^16, 47, 48^ miR-17 lentivirus construct was generated by inserting two copies of miR-17 precursor into the plv vector (Biosettia, CA, USA). RNA extraction and real-time PCR were performed as previously described,^16^ with actin or U6 as the correspondent normalization controls. Western blotting was performed as previously described.^16^ Supplementary Tables 1 and 2 list primers and antibodies utilized in this study.

### The generation of LUC constructs and LUC activity assay

DNA fragments of the mouse Tcf7l2 gene 3′UTR (wild type and the mutant, Figure 2i) were obtained by RT-PCR. DNA sequencing confirmed PCR products were then inserted into the pMir-Report (Ambion, Foster City, CA, USA) with the restriction enzymes SacI and MluI. To serve as a negative control, a non-related sequence was amplified from the coding sequence of the chicken versican G3 domain using two primers, chver10051SpeI and chver10350SacI, as previously documented.^47^

A dual-luciferase reporter system (Promega, Madison, WI, USA) was used to perform the luciferase activity assay. Briefly, 293 T cells were cultured on 12-well tissue culture plates at a density of 2 × 10^5^ cells per well. Cells were co-transfected with the luciferase reporter constructs, corresponding miRNA mimics and Renilla luciferase construct. After 24-h culture, transfected cells were lysed by 150 μl of passive lysis buffer. 30 μl of lysates were mixed with 50 μl of LAR II, and then firefly luciferase activity was measured by a luminometer. 50 μl of Stop & Glo reagent was added to the sample as the internal control.^34^

### Statistical analysis

Results are expressed as mean+S.E.M. Statistical analysis was performed by one-way ANOVA followed by the Newman–Keuls method for multiple comparisons using GraphPad Prism 5. Comparison between two sets of samples was analysed by Student's *t*-test. Statistical significance was determined at *P*<0.05.
