# Supplementary material for: Curcumin represses mouse 3T3-L1 cell adipogenic differentiation via inhibiting miR-17-5p and stimulating the Wnt signalling pathway effector Tcf7l2
Source: Cell Death Dis. 2017 Jan 19;8(1):e2559–. doi: 10.1038/cddis.2016.455 (PMC5386366; doi:10.1038/cddis.2016.455)
Supplement: Supplementary Information [file cddis2016455x1.pdf]

Materials and Methods

Mouse primary hepatocytes isolation: Male C57BL/6 mouse (6-8 weeks) were utilized for mouse primary hepatocyte preparation. As we have previously described, hepatic portal vein was cannulated with a 25G evacuated tube butterfly needle in the mice, and liver anterograde collagenase perfusion was conducted using a peristaltic pump [1]. Hepatocytes extracted from the digested liver were culture in 10% FBS containing DMEM (15 mmol/L HEPES, 25 mmol/L glucose, 1 mmol/L sodium lactate, 1% penicillin/streptomycin, 100 nmol/L dexamethasone). Cells were utilized for designated experiments within a 72 h time frame.

Reference

[1] L. Tian, K. Zeng, W. Shao, B.B. Yang, I.G. Fantus, J. Weng, T. Jin, Short-Term Curcumin Gavage Sensitizes Insulin Signaling in Dexamethasone-Treated C57BL/6 Mice, The Journal of nutrition 145(10) (2015) 2300-7.

Supporting Figure Legends

Figure S1. The effect of HFD feeding on the expression of hepatic miR-17 and other miR-17/92 cluster members. A) Mature miR-17-5p level. **B)** miR-17 precursor level. **C)** Other members of the cluster. N> or =3. HFD, High Fat Diet; miR-17, miR-17-5p.

Figure S2. The expression level of miR-17-5p in 3T3-L1 cells after miR-17 inhibition (A) or over-expression (B). N> or =3. CON, control.

Figure S3. The effect of miR-17 over-expression and inhibition on the levels of *Tcf7*l2 in mouse primary hepatocytes A-B) *Tcf7l2* mRNA level after miR-17 over-expression. **C-D)** *Tcf7l2* mRNA level after miR-17-5p inhibition. N> or =3. **, P<0.01; ***, P<0.001. CON, control nucleotides.

Figure S4. The effect of curcumin treatment on miR-17-5p and Tcf7l2 expression in rat primary adipocytes. A) qRT-PCR results of miR-17-5p in rat adipocytes with 6 h curcumin treatment. **B)** Detection of Tcf7l2 protein expression in rat adipocytes with and without 6 h curcumin treatment.

Figure S5. The effect of miR-17 inhibition and curcumin treatment on Rb2 expression in 3T3-L1 cells. A) *Rb2* mRNA levels after miR-17-5p inhibition. **B)** *Rb2* mRNA levels after miR-17 overexpression. **C)** *Rb2* mRNA levels after curcumin treatment for 6 h. **D)** HFD feeding did not change hepatic *Rb2* mRNA expression level. N> or =3. *, P<0.05. CON, control nucleotides; CUR, curcumin.

Figure S6. No substantial morphology change in 3T3-L1 cells received 2 or 10 µM curcumin treatment for 72 h.

Fig. S1

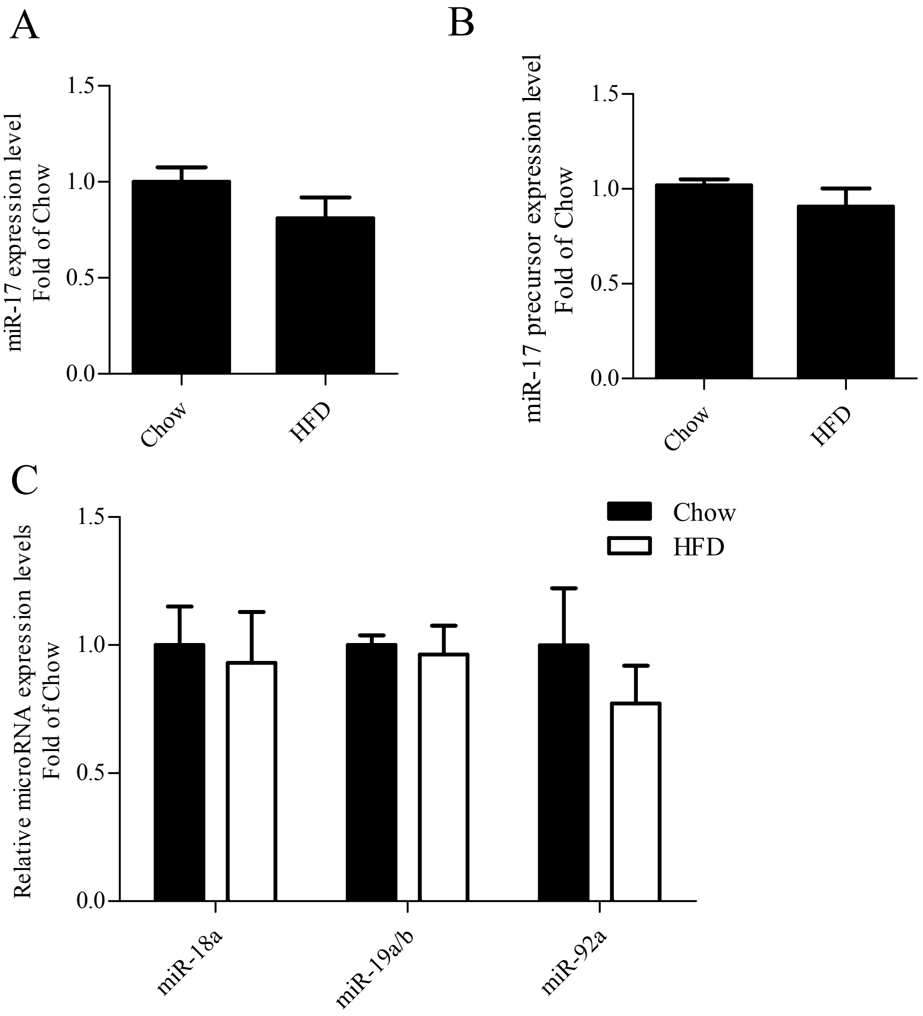
A
miR-17 expression level
Fold of Chow
1.5
1.0
0.5
0.0
Chow
HFD
B
miR-17 precursor expression level
Fold of Chow
1.5
1.0
0.5
0.0
Chow
HFD
C
Relative microRNA expression levels
Fold of Chow
1.5
1.0
0.5
0.0
Chow
HFD
miR-18a
miR-19a/b
miR-92a

Fig. S2

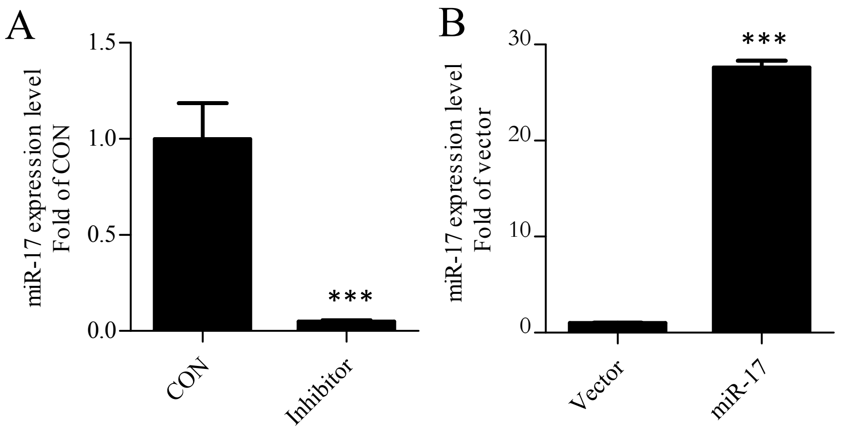

A
miR-17 expression level
Fold of CON
1.5
1.0
0.5
0.0
***
CON
Inhibitor
B
miR-17 expression level
Fold of vector
30
20
10
0
***
Vector
miR-17

Fig. S3

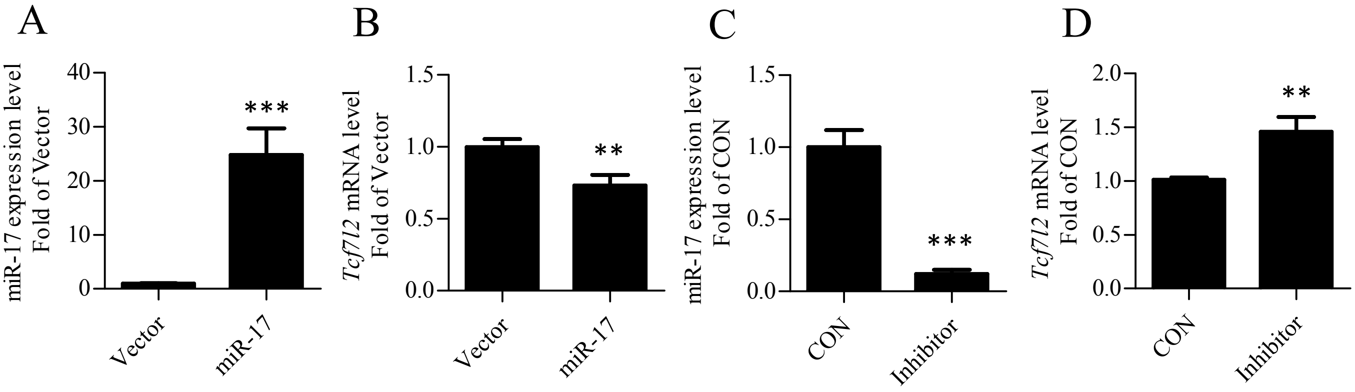
A
miR-17 expression level
Fold of Vector
40
30
20
10
0
***
Vector
miR-17
B
Tcf7l2 mRNA level
Fold of Vector
1.5
1.0
0.5
0.0
**
Vector
miR-17
C
miR-17 expression level
Fold of CON
1.5
1.0
0.5
0.0
***
CON
Inhibitor
D
Tcf7l2 mRNA level
Fold of CON
2.0
1.5
1.0
0.5
0.0
**
CON
Inhibitor

Fig. S4

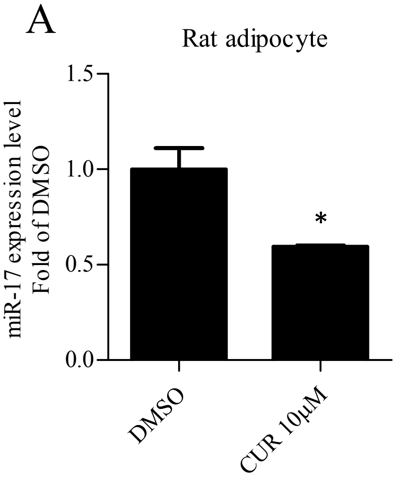
A
Rat adipocyte
miR-17 expression level
Fold of DMSO
1.5
1.0
0.5
0.0
*
DMSO
CUR 10μM

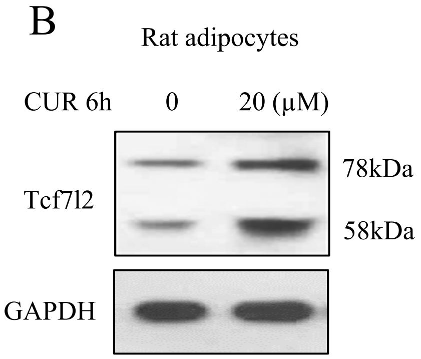
B
Rat adipocytes
CUR 6h
0
20 (μM)
Tcf7l2
78kDa
58kDa
GAPDH

Fig. S5

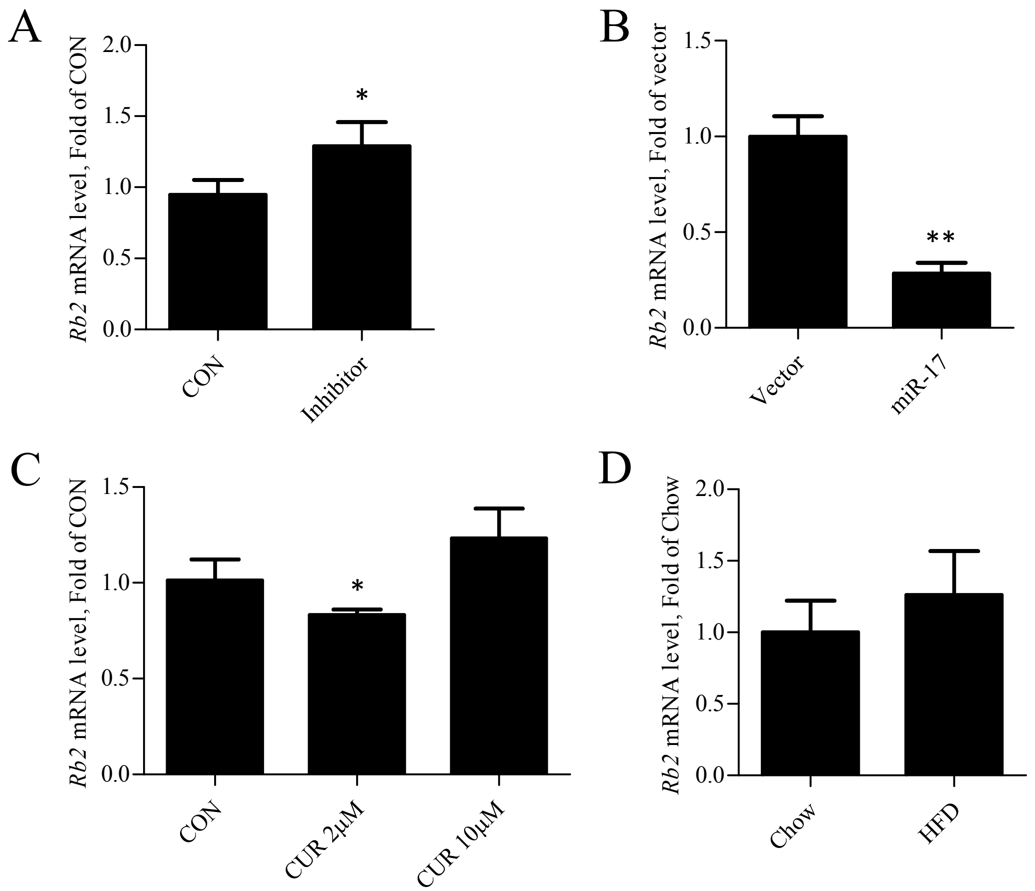
A
Rb2 mRNA level, Fold of CON
2.0
1.5
1.0
0.5
0.0
*
CON
Inhibitor
B
Rb2 mRNA level, Fold of vector
1.5
1.0
0.5
0.0
**
Vector
miR-17
C
Rb2 mRNA level, Fold of CON
1.5
1.0
0.5
0.0
*
CON
CUR 2μM
CUR 10μM
D
Rb2 mRNA level, Fold of Chow
2.0
1.5
1.0
0.5
0.0
Chow
HFD

Fig. S6

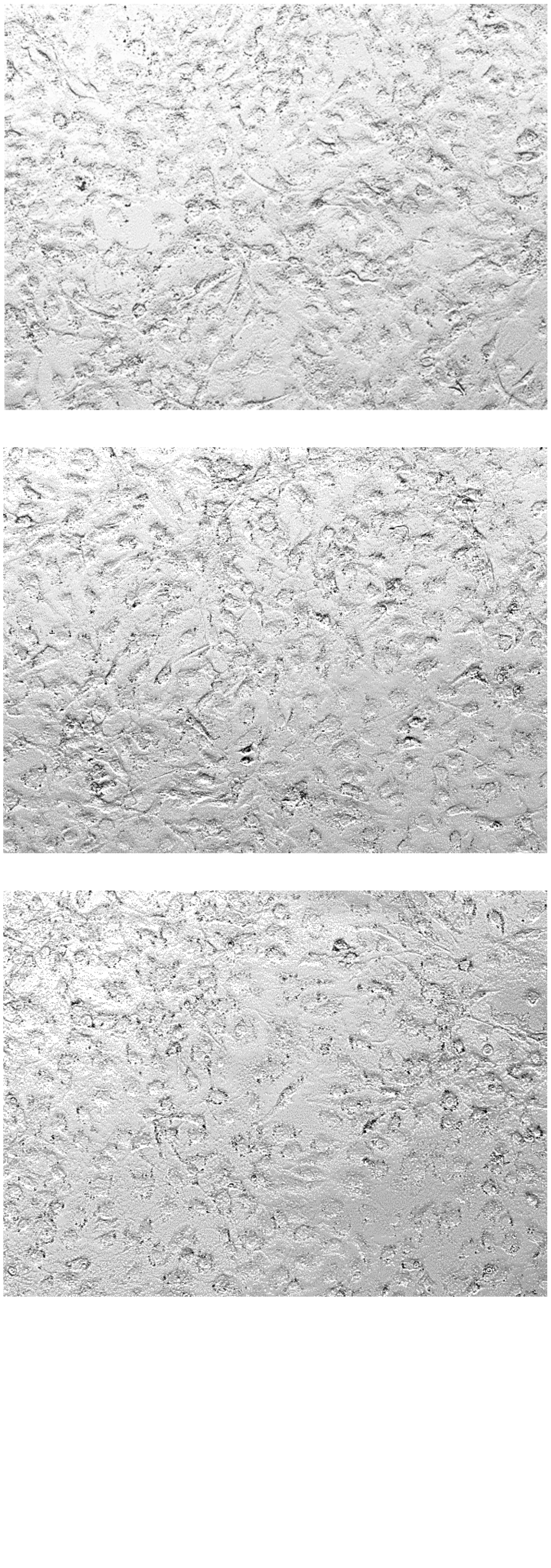

CON

CUR 2μM

CUR 10μM

Supplemental Table 1 Primers utilized in this study

Name	Forward	Reverse
miR-17	5'-CAAAGTGCTTACAGTGC-3'	5'-GTGCAGGGTCCGAGGT-3'
miR-17 precursor	5'-GTCAGAATAATGTCAAAGTGCTTAC-3'	5'-GTCACCATAATGCTACAAGTGCC-3'
miR-18a	5'-GCGTAAGGTGCATCTAGTGC-3'	5'-GTGCAGGGTCCGAGGT-3'
miR-19a	5'-CGGCGTGTGCAAATCTATGCAA-3'	5'-GTGCAGGGTCCGAGGT-3'
miR-92a	5'-GGCGTATTGCACTTGTCCC-3'	5'-GTGCAGGGTCCGAGGT-3'
U6	5'-GTGCTCGCTTCGGCAGCACATAT-3'	5'-CGCTTCACGAATTTGCGTGTCAT-3'
Tcf7	5'-AGGTCAGATGGGTTGGACTG-3'	5'-AGGGTGCACACTGGGTTTAG-3'
Tcf7l1	5'-GAGTGCGAAATCCCCAGTTA-3'	5'-ATGCATGGCTTCTTGCTCTT -3'
Tcf7l2	5'-AGGAACGACAGCTTCACATG-3'	5'-CGAAAGGCAAGGATTTAGGAAAC-3'
Rb2	5'-ATGCCACCTCAAAACCTAGAG-3'	5'-GGAGCTGTACCTGTCATACAATG-3'
Axin2	5'-TCCTGACCAAACAGACGAC-3'	5'-ACCTCTGCTGCCACAAAACT-3'
Actb	5'-TCATGAAGTGTGACGTTGACA-3'	5'-CCTAGAAGCATTGCGGTG-3'
Tcf7l2 3'UTR	5'-GGGAGCTC TTAGAATAGCTCCGCCTCCTCGTC-3'	5'-CCACGCGT AACTCCTTCTGGAGGGTGCTTCA-3'
Tcf7l2 3'UTR mutation	5'-GGGAGCTC TTAGAATAGCTCCGCCTCCTCGTC-3'	5'-CCACGCGT AACTCCTTCTGGAGGGTGCTTCA CTCGA GCTTC-3'
Cidea	5'-ATTTAAGAGACGCGGCTTTGGGACA-3'	5'-TTTGGTTGCTTGCAGACTGGGACAT-3'
C/EBPα	5'-CGAGGAGGACGAGGCGAAGCA-3'	5'-TGCGCAGGCGGTCATTGTCAC-3'
C/EBPβ	5'-CACCACGACTTCCTCTCCGACCTCT-3'	5'-GTACTCGTCGCTCAGCTTGTCCACC-3'
aP2	5'-TGAAAGAAGTGGGAGTGGGCTTTGC-3'	5'-CACCACCAGCTTGTCACCATCTCGT-3'
PPARγ	5'-CATAAAGTCCTTCCCGCTGA-3'	5'-ACCTCTTTGCTCTGCTCCTG-3'

Supplemental Table 2 Antibodies utilized in this study

Antibody Name	Catalog No.	Company
Tcf7l2	#2569	Cell Signaling Technology
Phospho-β-Catenin Ser675	#9567	Cell Signaling Technology
β-actin	sc69879	Santa Cruz Biotechnology
GAPDH	sc25778	Santa Cruz Biotechnology
HA	sc7392	Santa Cruz Biotechnology
